# Cross-continental national nutrition surveys: a narrative review

**DOI:** 10.1186/s40795-024-00868-4

**Published:** 2024-04-22

**Authors:** Areej A. Alkhaldy, Abeer M. Aljaadi, Abbe M. Mhd. Jalil, Doaa A. Alyoubi, Haneen H. Saleemani, Ruba H. Eid, Najlaa H. Almohmadi, Hala H. Al-Otaibi, Sarah M. Ajabnoor

**Affiliations:** 1 Department of Clinical Nutrition, Faculty of Applied Medical Sciences, King Abdulaziz University, P.O. Box 80215, Jeddah, 21589 Saudi Arabia; 2 Department of Clinical Nutrition, Faculty of Applied Medical Sciences, Umm Al-Qura University, Makkah, 21955 Saudi Arabia; 3 School of Nutrition & Dietetics, Faculty of Health Sciences, Universiti Sultan Zainal Abidin, Kuala Nerus, Terengganu 21300 Malaysia; 4 Department of Food and Nutrition Science, College of Agricultural and Food Science, King Faisal University, Al-Ahsa, 31982 Saudi Arabia; 5 Food, Nutrition and Lifestyle Unit, King Fahd Medical Research Centre, King Abdulaziz University, Jeddah, Saudi Arabia

**Keywords:** National nutrition survey, Diet survey, Nutrition surveillance, Monitoring

## Abstract

Many countries use national nutrition surveys (NNSs) to assess their population’s health and nutrition needs. However, NNS aims, approaches, tools, and measurements vary among countries. To date, there has been no review evaluating the NNSs and their practices worldwide to help conduct future NSSs. Therefore, this narrative review was conducted to 1) explore and tabulate current NNSs in five continents (Asia, Europe, Africa, North America, and Australia) and 2) help lay the foundation for establishing clear guidelines for future NNSs. The NNSs were identified using two approaches. First, an electronic database search was conducted with key terms in PubMed database. Second, a general web-based search on the survey webpages of governmental organizations was conducted using the same key terms to identify eligible surveys. The review included general adult population (≥ 18 years) with a cross-sectional design, excluding NNSs related to household-only surveys, specific age groups, or insufficient sample sizes. A total of 41 NNSs were identified in 37 countries across four continents: Asia (*n* = 15), Europe (*n* = 21), North America (*n* = 3), and Australia (*n *= 2). Broad differences between the surveys were identified, including survey purposes and designs, definitions of geographic areas and target groups, and dietary assessments. Currently, there are 26 ongoing NNSs, while 15 have ended. Among the ongoing NNSs, the cycles of the surveys were either at regular intervals (*n *= 8) or irregular intervals (*n* = 8). Of the 41 surveys, 24-h dietary recalls were used in 27 surveys, while only 6 surveys used diet diaries and 8 surveys relied on FFQs. Some surveys (*n* = 17) utilized multiple tools to assess dietary intake. Most of the surveys that assessed biochemical status (*n* = 12) focused on blood glucose, haemoglobin A1c (HbA1c), and lipid status, whereas some surveys (*n* = 6) tested for vitamin and mineral status in blood and/or urine samples. The review identified key characteristics, time frames, sampling methods, and dietary and physical assessment methods obtained from different surveys worldwide. The information organized in this review will be important for researchers, policymakers, and public health programme developers in developing and improving NNS.

**Supplementary Information:**

The online version contains supplementary material available at 10.1186/s40795-024-00868-4.

## Introduction

Noncommunicable diseases (NCDs), including cardiovascular diseases, cancers, diabetes, obesity, and chronic lung diseases, are the leading cause of death worldwide. The World Health Organization estimates that 41 million (74%) of the world's 57 million deaths each year are caused by NCDs, and approximately 17 millions of these deaths are premature (defined as those occurring among people aged between 30 and 70 years) [1, 2].

Prevention is economically more cost-effective than treating chronic diseases and their complications [3]. However, to effectively address the burden of NCDs, health care systems and public health services need to use integrated approaches as strategies. The US Centers for Disease Control and Prevention (CDC) reports four strategies: a) epidemiology and surveillance to monitor trends and inform programmes; b) environmental approaches that promote health and support healthy behaviours; c) health system interventions to improve the effective use of clinical and other preventive services; and d) community resources linked to clinical services that sustain improved management of chronic conditions [3].

For effective implementation of CDC strategies, targeted interventions and resource conservations, countries must rely on surveys and surveillance data. These data provide credible evidence to prioritize the intervention needs across diverse settings. National nutrition surveys (NNSs) are an important tool to obtain data on the nutrition and health status of the population [4–6].

Many countries worldwide use national nutrition and health surveys to assess health and nutritional needs, enable routine monitoring of the well-being of the population, facilitate the detection of unhealthy nutritional/health trends in a specific region of the country, plan prevention programs and formulate nutrition guidelines and public health policies [7–14]. In addition, information gathered by a nutrition surveillance and monitoring system can be used to detect changes in the nutritional status of a population and evaluate the effectiveness of nutrition programs [5, 15].

There have been a multitude of review papers conducted globally [5]. These review papers have delved into diverse aspects and concentrated on specific parameters of NNSs, including food consumption patterns [16, 17], physical activity [17], or the evaluation of specific blood biomarkers [18, 19]. Furthermore, certain review papers have encompassed on continent, such as Europe [5] or Asia [15]. While these review papers offer valuable insights, it is imperative to take into account the wider global context and examine NNSs worldwide. A comprehensive review of NNSs at the global level will serve as a platform to disseminate key information about the NNSs such as survey design, sampling techniques, data collection methods, and findings. This information is needed to advance and improve future surveys by using evidence-informed decision-making.

Therefore, this review explored and tabulated current national and international nutrition surveys conducted on five continents (Asia, Europe, Africa, North America and Australia) within the last 20 years. In addition, gathering information from different surveys worldwide allowed identification of the key characteristics, time frames, sampling methods, dietary and physical assessment methods, and medical data obtained. This may offer insights into potential challenges and concerns commonly associated with the execution of nationwide surveys aiming at nutritional assessment of the population. Moreover, this review will help researchers, policymakers, and public health developers when establishing or improving NNSs and provide recommendations for future NNSs in terms of surveys design, implementation, and reporting of such surveys.

## Methods

### NNS data acquisition

There are different sources for information of NNSs. According to previously published reviews [5, 16, 19], the most common information sources for NNSs include published reports in scientific databases, gray literature (i.e., official public websites of NNS), and actual personal contacts of survey’s investigators which may not be usable for some countries and for earlier surveys. Therefore, in this narrative review, two approaches were used to identify NNSs. First, an electronic database search was conducted in PubMEd database. The search was run for NNS with the first cycle occurring no later than 1946 till September 20, 2021. The following English key words were used for eligible surveys: (Survey* OR Surveillance*) AND (nutrition* OR diet* OR health* OR food*) AND (list of countries). This review only included surveys conducted on a national scale across the general adult (> 18 years) population with a prospective cross-sectional design that collected data at an individual level or at household and individual levels (household-only surveys such as Household Income and Expenditure Surveys or the National Household Food Acquisition, did not align with the aim of this review were excluded). The exclusion criteria included surveys that were related to food security, or had no assessment methods of any dietary intake (i.e., 24 h dietary recalls, food diaries, or quantitative/qualitative food frequency questionnaire), only included populations under 18 years such as children (2–12 years), adolescents (12–18 years) or one sex (e.g., surveys that only included children and women of reproductive age and pregnant), were published before 2000 (only for articles available in the electronic database), were not available in English, and had sample size that may not nationally representative of the whole population (*n* < 1000) [20]. Second, a general web-based search on survey webpages of governmental organizations was conducted using the same key terms to identify eligible surveys with no available published reports.

### Data extraction

The characteristics of the included surveys were extracted and tabulated. Five authors performed data extraction and four authors independently verified the accuracy of the extracted information. Two authors conducted independent evaluations to ensure the accuracy and completeness of all required data were incorporated. These included country name, full survey name with abbreviation, type of study design, level of survey (individual or household and individual), year of first and last cycle, interval of survey cycle, age of population, sampling method, sample size (i.e., average number per cycle for individuals and households), and response rate. Whenever possible, this review reported the response rate based on the last cycle of the survey. In addition, variables related to survey methodology and data collection were recorded, such as duration and period of data collection, methods used in data collection, type of data collected by all methods, and types of methods for collecting dietary intake data.

## Results

**Fig. 1:**
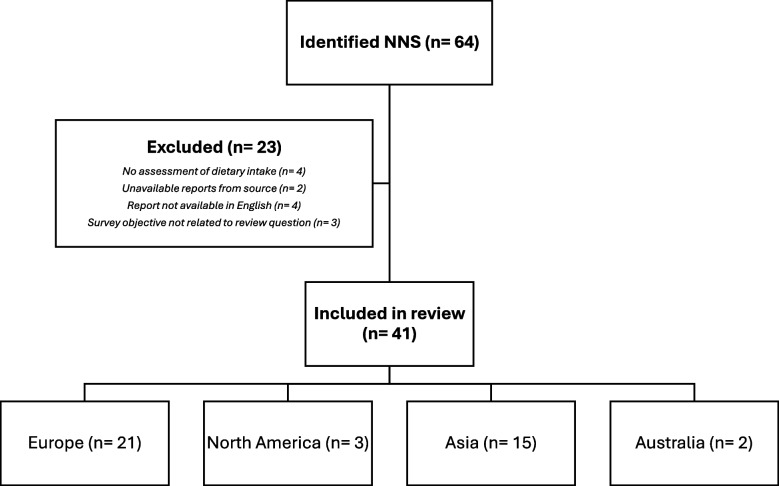
Summary of NNS included in the review

A total of 41 NNSs were identified in 37 countries across 4 continents (Table 1). A total of 23 surveys were excluded from the review due to the following reasons: no assessment of any dietary intake (*n =* 4), unavailable reports from source (*n =* 2), survey reports were not available in English (*n =* 4), survey objectives were not related to review question (*n =* 3), and inclusion of populations under 18 years and/or only women of reproductive age or pregnant (*n =* 10) (Fig. 1). The full names of the identified surveys, survey design, year, duration, population, sampling methods and collection are presented in Table 2.

**Table 1:** Identified national nutrition surveys according to continent (*n =* 41)

Country	Full survey name	Number
America		3
Canada	Canadian Health Measures Survey (CHMS) Canadian Community Health Survey—Nutrition (CCHS)	2
US	The National Health and Nutrition Examination Survey (NHANES)	1
Europe		21
UK	The National Diet and Nutrition Survey (NDNS)	1
Greece	Hellenic National Nutrition and Health (HNNHS) Greek National Diet and Health Survey (HYDRIA)	2
Poland	Multi-Centre National Population Health Examination Survey (WOBASZ)	1
Portugal	National Food and Physical Survey (IAN-AF)	1
Spain	National Food Survey on Adults, Elderly People and Pregnant Women (ENALIA 2)	1
Switzerland	The National Nutrition Survey (menuCH)	1
Turkey	Turkey Nutrition and Health Survey (TNHS)	1
Czech Republic	Czech National Food Consumption Survey (SISP)	1
Finland	The National FINDIET Survey	1
Hungary	Hungarian National Food Consumption Survey among Adults	1
Iceland	Icelandic National Nutrition Survey (NNS)	1
Austria	Austrian Nutrition Survey	1
Belgium	Belgium National Food Consumption Survey (BNFCS)	1
Denmark	Danish National Survey of Diet and Physical Activity (DANSDA)	1
France	French Nutrition and Health Survey (ESTEBAN)	1
Germany	German National Nutrition Survey (NVS)	1
Ireland	National Adult Nutrition Survey (NANS)	1
Italy	The Third Italian National Food Consumption Survey (INRAN-SCAI)	1
Netherlands	Dutch National Food Consumption Survey (DNFCS)	1
Asia		15
Russia	Russian Longitudinal Monitoring Survey (RLMS)	1
Japan	National Health and Nutrition Survey (NHNS)	1
Mongolia	Mongolia National Nutrition Survey (NNS)	1
South Korea	The Korea National Health and Nutrition Examination Survey (KNHANES)	1
China	China Health and Nutrition Survey (CHNS)	1
India	National Nutrition Monitoring Bureau (NNMB) Survey	1
Philippines	National Nutrition Survey (NNS)	1
Singapore	National Nutrition Survey (NNS) National Population Health Survey (NPHS)	2
Taiwan	Nutrition and Health Survey in Taiwan (NAHSIT)	1
Thailand	The Thai Food Consumption Survey (TFCS)	1
Saudi Arabia	Saudi Health Interview Survey (SHIS) National Survey of Health, Diet, Physical Activity and Supplements among Adults World Health Survey Saudi Arabia (KSAWHS)	3
Kuwait	Kuwait National Nutrition Survey (KNNS)	1
United Arab Emirates	UAE World Health Survey (UAEWHS)	1
Australia		2
Australia	National Health Survey (NHS)/National Nutrition and Physical Activity Survey (NNPAS)	1
New Zealand	New Zealand Adult Nutrition Survey (NZANS)	1

**Table 2:** Design of identified national nutrition surveys

Country	Survey name	Survey level^a^	Survey design	Year of first cycle	Year of last cycle	Interval of survey cycles	Population age	Sampling method	Sample size^a^	RR^a^ (%)	Ref
Canada	CHMS	Both	Ongoing	2007	2020	Every 2 y	3–79 y	Stratified three-stage	5000–10000 Individuals	50%—75%	[12]
Canada	CCHS	Individual	Ended	2004	2015	Not regular	≥ 1 y	Stratified three-stage	> 20,000 Individuals	50%—75%	[13]
US	NHANES	Both	Ongoing	1960	2022	Every 2 y	All groups	Complex, multistage, probability	5000–10000 Individuals	50%—75%	[8]
UK	NDNS	Both	Ongoing	2008	2019	Annually	≥ 1.5 y	Random sampling	1000–5000 Individuals	25%—45%	[4]
Greece	HNNHS	Both	Ended	2013/2015	NA	Not regular	Adults + children	Random stratified	1000–5000 Individuals	50%—75%	[21]
Greece	HYDRIA	Individual	Ended	2013/2014	NA	Not regular	≥ 18 y	Stratified two-stage random	1000–5000 Individuals	25%—50%	[22, 23]
Poland	WOBASZ	Both	Ongoing	2003/2005	2013/2014	Every 10 y	Adults	Stratified three-stage	5000–10000 Individuals	25%—50%	[24]
Portugal	IAN-AF	Individual	Ongoing	1980	2015/2016	Not regular	3 mo-84 y	Multistage	5000–10000 Individuals	25%—50%	[25, 26]
Spain	ENALIA 2	Individual	Ongoing	2012	2014/2015	Not regular	18–75 y	Random	1000–5000 Individuals	50%—75%	[27]
Switzerland	The National Nutrition Survey (menuCH)	Individual	Ended	2014/2015	NA	Not regular	18–75 y	Random three-stage	1000–5000 Individuals	< 25%	[28, 29]
Turkey	TNHS	Both	Ongoing	1974	2017	Every 5 y	All groups	Weighted, multistage, stratified cluster	5000–10000 Individuals; 15,000–20000 Households	> 75%	[30]
Russia	RLMS	Both	Ongoing	1994	2013/2014	Annually	All groups	Multistage probability	15,000–20000 Individuals; 5000–10000 Households	> 75%	[31, 32]
Czech Republic	SISP	Individual	Ended	1990	2010	Not regular	4–90 y	Systematic random	1000–5000 Individuals	25%—50%	[33, 34]
Finland	FINDIET	Individual	Ongoing	1972	2017	Every 5 y	18–74 y	Stratified random	1000–5000 Individuals	50%—75%	[35, 36]
Hungary	Hungarian National Food Consumption Survey among adults	Individual	Ended	2018/2020	NA	Not regular	10–74 y	Stratified random	1000–5000 Individuals	50%—75%	[37]
Iceland	Icelandic NNS	Individual	Ended	1990	2010/2011	Not regular	18–80 y	Random	1000–5000 Individuals	50%—75%	[38]
Austria	Austrian Nutrition Survey	Individual	Ongoing	1998	2018	Every 4 y	18–64 y	Multistage cluster	1000–5000 Individuals	NS	[39]
Belgium	BNFCS	Both	Ended	2004	2014/2015	Not regular	3–64 y	Multistage stratified	1000–5000 Individuals	NS	[40]
Denmark	DANSDA	Individual	Ongoing	1985	2011/2013	Not regular	4–75 y	Probability	5000–10000 Individuals	50%—75%	[41–43]
France	ESTEBAN	Individual	Ongoing	1993/1994	2014/2016	Not regular	6–74 y	Stratified random	1000–5000 Individuals	25%—50%	[44]
Germany	NVS	Both	Ongoing	1985	2007	Not regular	14–80 y	Stratified two-stage	15,000–20000 Individuals	25%—50%	[45, 46]
Ireland	NANS	Individual	Ended	2008/2010	NA	Not regular	≥ 18 y	Random	1000–5000 Individuals	50%—75%	[47, 48]
Italy	INRAN-SCAI	Individual	Ended	1980	2005/2006	Not regular	0.1–97.7 y	Multistage stratified sampling	1000–5000 Individuals	25%—50%	[14, 49]
Netherlands	DNFCS	Individual	Ongoing	2003	2019/2021	Not regular	1–79 y	Stratified	1000–5000 Individuals	50%—75%	[50]
Japan	NHNS	Both	Ongoing	1946	2019	Annually	All groups	Stratified random	5000–10000 Individuals; 1000–5000 Households	50%—75%	[6]
Mongolia	Mongolia NNS	Both	Ongoing	1992	2017	Not regular	15–49 y + children	Stratified cluster-randomized	5000–10000 Individuals; 1000–5000 Households	> 75%	[51]
South Korea	KNHANES	Both	Ongoing	1998	2019	Annually	≥ 1 y	Multistage clustered probability	10,000–15000 Individuals	> 75%	[52]
China	CHNS	Both	Ongoing	1989	2015	Not regular	Adults + children	Multistage random cluster	> 20,000 Individuals; 5000–10000 Households	NS	[53]
India	NNMB Survey	Both	Ongoing	1974	2015/2016	Every 5 y	≥ 20 y	Multistage stratified random	> 20,000 Individuals	> 75%	[54]
Philippines	NNS	Both	Ongoing	1978	2021	Every 5 y	All groups	Multistage stratified	> 20,000 Individuals; > 20,000 Households	> 75%	[9]
Singapore	NNS	Individual	Ongoing	1993	2018	Every 6 y	18–69 y	Stratified	< 1000 Individuals	50%—75%	[55]
Singapore	NPHS	Both	Ongoing	1992	2021/2022	Annually	18–79 y + children and seniors	Two-stage	5000–10000 Individuals; 5000–10000 Households	50%—75%	[55]
Taiwan	NAHSIT	Both	Ongoing	1980	2013/2016	Every 5 y	All groups	Multistage, stratified, clustered, probability	1000–5000 Individuals; 5000–10000 Households	50%—75%	[11]
Thailand	TFCS	Individual	Ended	2004/2005	NA	Not regular	≥ 3 y	Stratified three-stage	15,000–20000 Individuals	> 75%	[56]
Saudi Arabia	SHIS	Both	Ended	2013	NA	Not regular	≥ 15 y	Multistage stratified probability	10,000–15000 Individuals; 10,000–15000 Households	> 75%	[57]
Saudi Arabia	National Survey of Health, Diet, Physical Activity and Supplements among adults	Individual	Ended	2018	NA	Not regular	≥ 18 y	Stratified random	1000–5000 Individuals	NS	[58]
Saudi Arabia	KSAWHS	Both	Ongoing	2010	2019	Every 5 y	≥ 15 y	Stratified three-stage, probability proportional to population size	5000–10000 Individuals; 5000–10000 Households	> 75%	[59]
Kuwait	KNNS	Both	Ended	2008/2009	NA	Not regular	3–86 y	Stratified cluster	1000–5000 Individuals; 1000–5000 Households	< 25%	[60]
United Arab Emirates	UAEWHS	Both	Ongoing	2003	2018	Every 5 y	Adults + children + pregnant women	Multistage cluster	5000–10000 Individuals; 10,000–15000 Households	> 75%	[61]
Australia	NHS/NNPAS	Both	Ongoing	1989/1990	2020/2021	Every 2 y	> 2 y	Multistage random	10,000–15000 Individuals; 5000–10000 Households	> 75%	[62]
New Zealand	NZANS	Both	Ended	1977	2008/2009	Not regular	≥ 15 y	Multistage, stratified, probability proportional to size sample design	1000–5000 Individuals	NS	[63]

*Abbreviations: RR* response rate

^a^Survey level: only surveys conducted among individuals or individuals and households (both). Sample size: reported as the average number per cycle. RR%: response rate is based on the average rate of overall completion of major elements of the survey

The objectives of surveys differed among the countries (Supplementary Table). There were nine objectives focused on health and status and diseases. Twenty-two surveys were conducted to evaluate and collect information regarding nutrition status, food consumption, and/or nutrient intake, with only six surveys of them indicated physical activities measures in their objectives. Only, ten surveys objectives indicated both health and nutrition/diet measures without physical activities.

### Survey design and sampling

All included surveys used a cross-sectional study design, except the China Health and Nutrition Survey (CHNS) [53], which is an ongoing open cohort survey (Table 2). Different methods for sampling were used by the NNSs. Nearly half (*n =* 18) of the included NNSs in this review used the multistage sampling method, which typically used a combination of stratified or cluster sampling and simple random sampling. Stratified sampling was also commonly used by NNSs (*n =* 17). However, very few surveys used simple random sampling (*n =* 5) or systematic random sampling (*n =* 1).

Over 50% of the surveys (*n =* 23) were conducted at both the individual and household levels, and the rest (*n =* 18) were conducted only at the individual level. No household-only surveys were included for the purpose of this review.

The number of individuals or households included in these surveys differed between surveys and between cycles of the same survey. Overall, the number of participants ranged from 1,000 to over 20,000. Great variability was observed in the overall response rates between surveys, ranging from 15 to 100% (Table 2). Switzerland had the lowest response rate (15%) in its National Nutrition Survey (menuCH) [28, 29], whereas India had the highest response rate (100%) in its National Nutrition Monitoring Bureau (NNMB) survey [54]. The highest response rates were generally reported by surveys in Asian countries [15], except for Kuwait, which had a response rate of 24% [60]. Notably, five surveys did not state their response rates.

### Survey populations and collection methods

The majority of identified surveys (*n =* 26) recruited more than one age group (i.e., children or adolescents or both in addition to adults). However, four surveys included individuals ≥ 15 years old, ten included participants ≥ 18 years old, and one survey included participants ≥ 20 years old. All surveys included both males and females.

All NNSs (*n =* 40), except the Italian Survey [14, 49] conducted interviews in a variety of formats including in-person interviews, phone interviews, computer-assisted telephone interviews (CATI), and computer-assisted in-person interviews (CAPI) to collect sociodemographic, health, lifestyle, physical activity, and dietary data. National nutrition/health surveys commonly took from two months to over one year for data collection. Data collection in waves of the same survey could overlap when the target population was different. Different methods were used for data collection, with interviews (in-person, phone, CATI, and/or CAPI) being the primary method to collect sociodemographic, health, and lifestyle data. However, most surveys used multiple methods for data collection: interviews, physical exams, and blood samples. Different types of interviews were used for data collection (e.g., home interview, phone interview, CAPI, or CATI).

Thirty-seven surveys included a physical exam to assess anthropometric and physiological measurements, and 23 surveys collected blood or urine samples to assess nutritional status. Biological data were either collected at a mobile examination centre or at participants’ homes by certified laboratory professionals. Physical exams were carried out either at the study participant’s home, at a health centre or at a mobile exam centre. Several surveys recorded physical measurements and/or collected blood samples in a subset, which was more practical and depended on the resources available.

Anthropometric measurements were self-reported by all participants in some surveys, such as those from Italy [14, 49] and one from Saudi Arabia [58]. However, some surveys used self-reported weight and height and measured them in a subsample for validation purposes. All 41 surveys assessed dietary intake but used different methods. Some surveys explored dietary habits/practices, the use of dietary supplements, food insecurity, eating practices, and food purchasing.

### Survey data

There were four main data types in the reviewed surveys: biological, anthropometric, physical activity, and dietary intake data.

#### Biological data

A total of 23 surveys from 22 different countries collected blood and/or urine samples from a subset of the population. Numerous surveys (*n =* 12) focused on nutritional markers, including blood glucose, haemoglobin A1c (HbA1c), and lipid status. Some surveys (*n =* 6) also tested for vitamin and mineral status in blood and/or urine samples. Other surveys investigated more specific biomarkers, such as renal/liver function in Taiwan [11]*,* genetic biomarkers in Poland [24], thyroid hormone in Greece [21–23], and infection markers in Canada [12, 13]. In India, researchers used minimally invasive finger prick tests rather than collecting venous blood samples to test for blood glucose and haemoglobin status [54]. In the Philippines and Finland, participating in the study required fasting for a minimum of 4 h to test for lipid profile and inflammatory markers, and fasting for 10 h for lipid profile and blood glucose determination [9, 35]. In Australia, participants who provided blood and urine samples were given a grocery voucher as compensation [62].

Eighteen of the 41 surveys had missing information, that was not accessible through online sources or did not report any biological data. Age ranges for biological data collection were not uniform. In a survey from Saudi Arabia, data were collected from participants aged 15 years and older [57, 59], while in a Japanese survey, only participants 20 years and older were included for biological data collection [6]. In a survey from China, biological data were not only limited to blood and urine samples; researchers also collected faeces, toenails and buccal swabs [53].

#### Anthropometric data

Thirty-eight surveys included a physical anthropometric measurement. Height and weight were measured using a stadiometer and weighing scale (analogue or digital) according to standard procedures at the study participants’ homes, at a health centre or at a mobile exam centre. Three studies relied on self-reported measurements of height and body weight. Body mass index (BMI) was calculated from height and weight as body weight (kg) divided by height (m) x height (m). While most surveys collected multiple measurements, one-third of the studies (*n =* 13) relied on measuring anthropometrics for nutritional status. The instrumentation used differed across studies, with SECA, Tanita and OMRON being the most commonly used brands for height and weight to the nearest 0.1 to 1 cm and 0.1 kg, respectively. Waist and hip circumference were measured to the nearest 0.1 cm using nonelastic plastic tape, and one study used nonelastic aluminium tape to reduce erratic measurement. Most weight measurements were performed with light clothing and shoes removed. WHO cut-off points were used to classify BMI categories for the USA and Europe, including the UK studies [4, 5, 8], while a specific WHO BMI for Asians was used by most Asian surveys [15].

#### Dietary data

Different types of dietary data were collected by different surveys (Table 3). All 41 surveys assessed dietary intake. However, some surveys also assessed dietary habits, the use of dietary supplements, food insecurity, eating practices, and food purchasing. The most popular tool used for food intake research worldwide was the 24-h recall tool which involves a structured interview to recall and report all food and beverages individual consumed during the past 24-h. Twenty-seven surveys used 24-h dietary recalls to assess dietary intake. Only six surveys used diet diaries, while eight surveys relied on food frequency questionnaires (FFQs) alone to assess dietary intake. Some surveys (*n =* 17) used more than one tool to assess dietary intake. There was variation in the collection methods for the 24-h recalls. For example, in India, the NNMB survey collected the 24-h recalls only from a subsample [54]. Overall, the period for collected food recalls ranged between 1 and 7 days. A total of 18 surveys collected multiple 24-h recalls (with more than one a day). The duration between the 24-h recalls varied across surveys with multiple recalls. Some surveys collected 24-h recalls on consecutive days, while others collected repeated 24-h recalls between 8 and 15 days apart or even 1 to 6 months apart. The New Zealand Adult Nutrition Survey (NZANS) had a random subsample complete the second 24-h recall within a month of the first recall [63]. For surveys with food diaries, the range of diary collection days was 3 to 7 days. The Danish National Survey of Diet and Physical Activity (DANSDA) recorded the highest number of days, using 7-day web-based food diaries [41–43]. Picture albums and sometimes the Global Picture Book for portion sizes (standardized measurements or household units) were used in Taiwan, Finland, Spain, Thailand and Iceland [11, 27, 35, 38, 56]

**Table 3:** Data collection in identified national nutrition surveys

Country	Survey name	Duration of data collection	Data collection methods	Type of data collected during physical exam	Type of dietary data	Methods for collecting dietary intake data
Canada	CHMS	2 y	Home interview, physical exam, and laboratory tests	Anthropometrics, physiological measurements, blood and urine samples	Dietary intake and habits	FFQ
Canada	CCHS	1 y	CAPI, CATI, and physical exam	Anthropometrics	Dietary intake, habits, supplements, and food security	24-h recall
US	NHANES	2 y	Home and phone interview, physical exam, and laboratory tests	Anthropometrics, physiological measurements, imaging, dental check, blood and urine samples	Dietary intake, habits, and food security	2-day 24-h recall
UK	NDNS	2 y	Interview, physical exam, and laboratory tests	Anthropometrics, physiological measurements, blood and urine samples	Dietary intake	4-day food diary
Greece	HNNHS	1.5 y	CAPI, physical exam, and laboratory tests	Anthropometrics, physiological measurements, and blood samples	Dietary intake and eating practices	2-day 24-h recall + FPQ
Greece	HYDRIA	1.5 y	In-person interview, physical exam, and laboratory tests	Anthropometrics, physiological measurements, and blood samples	Dietary intake	2-day 24-h recall + FPQ + Eating out choices questionnaire
Poland	WOBASZ	1 y	In-person interview, physical exam, and laboratory tests	Anthropometrics, physiological measurements, and blood samples	Dietary intake	24-h recall + FFQ
Portugal	IAN-AF	1 y	In-person interview, CAPI, and physical exam	Anthropometrics	Dietary intake, habits, supplements, and food insecurity	2-day 24-h recall + FFQ
Spain	ENALIA 2	1 y	Home interview, CATI, and physical exam	Anthropometrics	Dietary intake and supplement use	2-day 24-h recall + FPQ + FFQ
Switzerland	The National Nutrition Survey (menuCH)	1 y	CAPI, CATI, and physical exam	Anthropometrics	Dietary intake	2-day 24-h recall
Turkey	TNHS	1 y	Home interview, physical exam, and laboratory tests	Anthropometrics, physiological measurements, blood and urine samples	Dietary intake, habits, supplements, and food purchasing	1-day 24-h recall + FFQ
Russia	RLMS	4–5 mo	In-person interview and physical exam	Anthropometrics	Dietary intake	1-day 24-h recall
Czech Republic	SISP	1 y	Home interview, physical exam, and laboratory tests	Anthropometrics, physiological measurements, and blood samples	Dietary intake	2-day 24-h recall
Finland	FINDIET	10 mo	CAPI, CATI, physical exam, and laboratory tests	Anthropometrics, physiological measurements, and blood samples	Dietary intake and supplement use	2-day 24-h recall + FPQ
Hungary	Hungarian National Food Consumption Survey among adults	2 y	Home interview, CATI, and physical exam	Anthropometrics	Dietary intake and habits	2-day 24-h recall + FPQ
Iceland	Icelandic NNS	1 y	Phone interview	None	Dietary intake and habits	2-day 24-h recall + FFQ
Austria	Austrian Nutrition Survey	2 y	CAPI, CATI, self-administered/online questionnaire, and physical exam	Anthropometrics	Dietary intake	2-day 24-h recall + FFQ
Belgium	BNFCS	NS	Oral interview and physical exam	Anthropometrics	Dietary intake	2-day 24-h recall + FFQ
Denmark	DANSDA	NS	In-person interview, physical exam, and laboratory tests	Anthropometrics, physiological measurements, and blood samples	Dietary intake	7-day food diary
France	ESTEBAN	2 y	Personal interview, physical exam, and laboratory tests	Anthropometrics, physiological measurements, and blood samples	Dietary intake	3-day 24-h recall
Germany	NVS	NS	CAPI, self-administered questionnaire, and physical exam	Anthropometrics	Dietary intake	2-day 24-h recall + FFQ
Ireland	NANS	NS	Research visit, physical exam, and laboratory tests	Anthropometrics, physiological measurements, blood and urine samples	Dietary intake	4-day food diary
Italy	INRAN-SCAI	1.2 y	Only self-recorded food diary	Anthropometrics (self-reported)	Dietary intake	3-day food diary
Netherlands	DNFCS	4 y	Home interview and physical exam	Anthropometrics	Dietary intake and habits	2-day 24-h recall
Japan	NHNS	1 mo	In-person interview and physical exam, and laboratory tests	Anthropometrics, physiological measurements, and blood samples	Dietary intake, habits, and food purchasing	3-day food diary
Mongolia	Mongolia NNS	NS	In-person interview, physical exam, and laboratory tests	Anthropometrics, physiological measurements, blood and urine samples	Dietary intake, food security, and supplement use	24-h recall + FFQ
South Korea	KNHANES	1 y	In-person interviews, physical exam, and laboratory tests	Anthropometrics, physiological measurements, imaging, dental check, blood and urine samples	Dietary intake, habits, food security, and supplement use	24-h recall + FFQ
China	CHNS	NS	In-person interviews, physical exam, clinical and laboratory tests	Anthropometrics, physiological measurements, blood, urine, faeces and toenail samples and buccal swabs	Dietary intake	3-day food record + FFQ
India	NNMB Survey	2 y	In-person interview, physical exam, and laboratory tests	Anthropometrics, physiological measurements, and blood samples	Dietary intake	24-h recall
Philippines	NNS	4–10 mo	Home interview, physical exam, clinical and laboratory tests	Anthropometrics, physiological measurements, and blood samples	Dietary intake and food insecurity	2-day 24-h recall
Singapore	NNS	5 mo	In-person interview and laboratory tests	Urine samples	Dietary intake and practices	DPQ + FFQ
Singapore	NPHS	1 y	Home interview, physical exam, and laboratory tests	Anthropometrics, physiological measurements, blood and urine samples	Dietary intake and habits	FFQ
Taiwan	NAHSIT	NS	In-person interview, physical exam, and laboratory tests	Body composition, blood and urine samples	Dietary intake and habits	FFQ
Thailand	TFCS	1 y	Home interview and a physical exam	Anthropometrics	Dietary intake and habits	24-h recall + FFQ
Saudi Arabia	SHIS	3 mo	Home interview, physical exam, and laboratory tests	Anthropometrics, physiological measurements, and blood samples	Dietary intake	FFQ
Saudi Arabia	National Survey of Health, Diet, Physical Activity and Supplements among adults	6 mo	CAPI and CATI	Anthropometrics (self-reported)	Dietary intake, habits, and shopping practices	FFQ
Saudi Arabia	KSAWHS	NS	CAPI, physical exam, and laboratory tests	Anthropometrics, physiological measurements, and blood samples	Dietary intake of selected items	FFQ
Kuwait	KNNS	1.5 y	In-person interview, physical exam, and laboratory tests	Anthropometrics, physiological measurements, and blood samples	Dietary intake and habits	24-h recall
United Arab Emirates	UAEWHS	6 mo	CAPI, physical exam, and laboratory tests	Anthropometrics, physiological measurements, and blood samples	Dietary intake of selected items	FFQ
Australia	NHS/NNPAS	1 y	In-person and phone interview, physical exam, and laboratory tests	Anthropometrics, physiological measurements, blood and urine samples	Dietary intake, habits, and supplements	2-day 24-h recall
New Zealand	NZANS	1 y	In-person interview, physical exam, and laboratory tests	Anthropometrics, physiological measurements, blood and urine samples	Dietary intake, habits and food security	1–2-day 24-h recall

*Abbreviations: FFQ* food frequency questionnaire, *CAPI* computer-assisted personal interview, *CATI* computer-assisted telephone interview, *FPQ* food propensity questionnaire, *DPQ* dietary practices questionnaire, *h* hour, *mo* month, *y* year, *NS* not stated

#### Physical activity data

Surveys from Europe, Greece, Finland, Denmark, Spain, Iceland, Portugal, Belgium, France, and Ireland included questions and gathered data on the topic of physical activity [21–23, 25–27, 35, 38, 40–42, 44, 47, 48]. Asian countries, namely, Japan, China, Saudi Arabia, South Korea, the UAE, and Singapore estimated physical activity in their surveys [6, 52, 53, 55, 58, 61]. Eighteen studies estimated physical activity qualitatively using the International Physical Activity Questionnaire (IPAQ), Global Physical Activity Questionnaire (GPAQ) or other ‘self-developed’ questionnaires. Two studies objectively measured physical activity using a pedometer and accelerometer (*n =* 1). Three studies used a combination of questionnaires and pedometers. Two studies did not record physical activity, but instead, a physical activity level (PAL) of 1.5 was used to estimate basal metabolic rates. Two surveys estimated physical inactivity (pedometers and accelerometers) instead of physical activity. The remaining 13 surveys did not record physical activity.

#### Survey administration

National health surveys varied between countries with respect to survey initiation, the number of cycles conducted, and the frequency of survey cycles. Japan started their National Health and Nutrition Survey (NHNS) as early as 1946 and continued for decades [6]. The National Health and Nutrition Examination Survey (NHANES) in the US started in the 1960s [8], and surveys in the Philippines, India, Finland, New Zealand, the United Arab Emirates, and Turkey were initiated in the 1970s [10, 30, 35, 54, 61, 63]. Sixty-one percent (*n* = 27) of national surveys were considered continuous/ongoing surveys, as more than one cycle was conducted. These comprised surveys on all continents: Asia (including Russia, *n* = 12), Europe (*n* = 12), North America (*n* = 2), and Australia (*n* = 1).

Currently, there are 26 ongoing NNSs, while 15 have ended. Among the ongoing NNSs, the cycles of the surveys were either at regular intervals (*n* = 18) or irregular intervals (*n* = 8). Surveys conducted at regular intervals varied in the frequency of cycles, and the frequency sometimes changed throughout the years. For example, some surveys switched from a 5-year interval to a 3-year interval or from a 3-year interval to a 1-year interval. Our data showed that five ongoing surveys are currently conducted annually, and five are conducted every 2-3 years. Others opted for a 4- to 6-year interval (*n* = 7).

## Discussion

This review detailed the initial findings of 41 NNSs across five continents (Asia, Europe, Africa, North America and Australia) within the last 20 years, providing important baseline data to researchers, policymakers, and public health programme developers and laying the foundation for future NNSs. Overall, some broad differences between surveys were identified, including survey purposes and designs, definitions of geographic areas and target groups, and dietary assessments.

The objectives of the reviewed surveys varied between countries. Some surveys focused on health and nutrition, such as Hellenic National nutrition and Health (HNNHS), NHANES, and NHNS [6, 8, 21]. Other surveys, such as the National Diet and Nutrition Survey (NDNS, in the UK), German National Nutrition Survey (NVS), and Icelandic National Nutrition Survey (NNS), were conducted only to evaluate nutritional status [4, 45, 64]. Some surveys, such as the DANSDA and National Nutrition and Physical Activity Survey (NNPAS, in Australia), included examinations of physical activity [41, 62]. The overall purpose of the surveys depended on the priorities and needs of survey administrators and governmental departments [65]. Differences in approaches may also have been due to the financial, physical and human resources available in each country, as well as their experiences in conducting national surveys. All surveys provided justification for obtaining some health statistics.

The variation between countries in the purpose and methodology of surveys limits intercountry comparisons and the opportunity to provide recommendations. Therefore, it would be helpful to target future efforts at standardizing the purpose of conducting such surveys and filling knowledge gaps for countries that have no surveys to increase the information available for evidence-based policy planning across population groups worldwide. For example, in countries such as Canada [12, 13], Greece [21, 22], and Saudi Arabia [57–59], multiple surveys were identified with different purposes and were carried out by different administrations/groups. The results of these surveys were challenging to compare, as they differed in their purposes, type of data collected, and tools used. There is a great need for each country to assign the administration and management of their NNSs to one governmental health department to reduce overlapping work and variation in reporting, findings, and numbers of surveys. This will also facilitate data accessibility and subsequent analyses by researchers from all around the country, which maximizes the benefit of surveys.

In addition, the variation between countries in age groups, dietary tools, and time frames make it challenging to draw direct comparisons and produce comparable results. For example, different age groups vary in their dietary requirement and nutritional status of each age group. Different dietary tools also make it difficult to compare food consumption and nutrient intake between countries. The dietary tools range from 24-h dietary recalls, food diaries to food frequency questionnaires and each dietary tool have different types of questions, portion size, and reporting period, all of which could influence accuracy and data interpretation. Time frames of the surveys could also impact the comparison between countries. The differences in time frames could influence the ability to capture product reformulations and health changes. Therefore, it is important to carefully interpret survey data, taking into consideration age groups, dietary tools, and survey time frame.

A cross-sectional design was used for all surveys except the CHNS, which is an ongoing open cohort survey [53]. The cross-sectional design provides a snapshot of diet and related behaviours for a particular group of individuals at a particular point in time and requires minimal costs and a short amount of time to perform. Therefore, with repeated cross-sectional surveys, it is possible to pool data from individual survey waves/years, such as the NDNS [4]. This could help to increase the sample size and could be a means of exploring possible differences in nutrition across survey waves. However, the majority of surveys captured a different group of individuals at each wave of the repeated cross-sectional survey, which is helpful in assessing current diet-related behaviours but not for tracking changes in the same group of individuals over time. Therefore, for future NNSs, it is important for survey administrators/governmental departments to consider whether using the same or different groups when conducting their surveys will achieve their aims successfully and provide national recommendations that help their population.

All NNSs used probability sampling methods, which included simple random sampling, stratified sampling, cluster sampling, multistage sampling, and systematic random sampling. Probability sampling techniques provide the main advantage of ensuring that the sample selected is representative of the population. It is recommended by UNICEF to use multistage sampling methods when targeting national-level indicators. Nevertheless, for some countries, using an efficient sampling method with a feasible sample size for future NNSs is crucial for generating generalizable findings at the national level [66].

A high response rate is critical for NNSs. Overall, the response rates ranged between 15 and 100%. For surveys with multiple cycles, there was variation in the response rates between cycles as well. Different factors could affect the response rate for NNSs. NNSs are comprehensive and could be associated with a high participant burden. Such surveys require the collection of many details and require the participant to attend a health centre or clinic to provide blood or urine samples. Survey administrators may be encouraged to give study participants more information (e.g., dietary feedback) as a thank you gift, which aids in increasing participation [20]. Other NNSs may use other methods to enhance participation (e.g., providing payment vouchers). However, the methods for enhancing participation in all included NNSs were not reported in this review.

Surveys that were ongoing tended to have higher response rates than those conducted once (mean of 60% vs. 54%), but this needs a more thorough analysis. We expect that regularly conducting national surveys will facilitate valid comparisons of data between cycles and the identification of trends in a population’s dietary habits and health indicators within the country. The ability to detect these trends early provides opportunities to promote and support positive dietary habits and to tackle health issues earlier.

Most NNSs conducted among adults also included other age groups. Data from surveys with different age groups are more comprehensive and provide a larger picture of the country’s nutritional status and trends, which help in designing the basis of public nutrition guidelines. Given that NNSs are difficult to implement, future surveys in countries without experience in NNSs may be developed in a simple way by focusing on one age group. However, if the country prefers a comprehensive and representative sample across different age groups is important for obtaining a holistic understanding of the nutritional status and dietary patterns within their population, practical considerations such as available budgets and resources should also be taken into account.

Other major considerations are the methods of selection of geographical areas and target groups. Most surveys have a map of the selected area with clear justification of their choice/selection. However, different geographical area selection methods were used across the surveys. All reviewed surveys were nationally representative and focused on regions of interest to survey administrators. In addition, target groups such as males, females, middle-aged adults, or middle-income groups were aligned to the interests of the administrators/institutions responsible for the surveys. While no ideal sampling method exists for national surveys, selecting a representative sample for the country is critical, as conclusions drawn from the survey are meant to be generalized to the whole population of interest. Recently, most surveys have used technology such as Geographic Information System to identify sampling areas. In addition, sampling techniques need to be country-dependent based on what is suitable for the geography, population, and available resources [66]. It is recommended by UNICEF to use multistage sampling methods when targeting national-level indicators [67].

Dietary intake data are obtained by 24-h recall, dietary records, or FFQs at individual and household levels. The collection of dietary data differed between surveys across the captured countries which yielded different dietary findings. Therefore, it is important to consider these variations when interpreting survey data as some surveys focused on food groups to obtain information about dietary guidelines and adherence to dietary recommendation, while others focused on nutrients and their associations with deficiency. Further reviews of dietary survey methods and NNSs need to be consider in future studies.

Many surveys used multiple tools to collect food intake data. However, the most common methods of collecting dietary intake data were the 24-h recall as well as food diaries, which were collected over multiple days, as opposed to relying on FFQs alone. Although 24-h recalls are known for underreporting [68] and may not capture foods consumed infrequently, such as fish oil and seasonal foods, their increased use could reflect their advantage in being less onerous for respondents and potentially providing more consistent results across all age and sex groups compared with other methods [69]. Unlike the single 24-h recall method, national surveys that use FFQs are valuable, as FFQs are usually cost-effective, easy to administer, less complicated, and are able to evaluate the average individual’s usual diet over a long-term period, with the added benefit of larger sample sizes [66]. However, the disadvantages of using FFQs include over-estimation of intake, lack of portion size estimation, and rank individuals based on their relative intake of nutrients and foods rather than providing absolute intake. Therefore, combining various approaches can offer a more comprehensive understanding of individuals' dietary habits and nutrient intake [70]. Some countries, such as the Philippines and Greece, used FFQs to overcome variation when data from two days of intake were collected [9, 23]. Differences in dietary intake assessment methodologies between countries may be a limiting factor when making intercountry comparisons of nutrient intake. This could be due to the lack of complete national food composition databases or the limited resources to train labour for performing the 24-h recall.

In general, the lack of completeness of national food composition databases and classification software/systems is a considerable limitation worldwide [71]. For example, few food composition databases are regularly updated to include new or reformulated products, which could introduce possible error in the energy and nutrient content information of foods and hence the reported intakes [72]. In addition, Asian countries, for example, have a variety of mixed dishes with many ingredients that are often shared among groups of people. This makes it very difficult to estimate the amounts of foods and ingredients consumed at an individual level in Asian countries [15]. Therefore, the improvement of current dietary assessment methods and development of new approaches, such as using technology-based dietary assessment and data imputation and modelling are needed to obtain dietary intake data in NNSs. To establish an accurate NNS, diet evaluation tools such as dietary reference intakes (DRIs), dietary guidelines, food guidance systems, and a food composition database specific to particular countries are needed.

This comprehensive review provides a valuable information related to NNSs in all five continents. Despite the considerable efforts made to achieve a comprehensive overview, it is acknowledged that some surveys may have been overlooked. For example, this review excluded non-English language NNSs which may limit the representation of specific populations and cultural contexts. In addition, the present review is classified as a narrative review, rather than a systematic review, as the primary sources of information on NNSs, such as details on sampling, instruments, and staff training, are reports, information on public agency websites, and personal communications, all of which are considered grey literature. These sources of information are often not indexed in scientific databases, making it challenging to obtain them using reproducible search strategies. As a result, narrative reviews may be criticized for their limited reproducibility. Given the aforementioned limitations and the purpose of this review, the two-step approach, which involved using both available literature and a general web-based search on survey webpages of governmental organizations, was conducted using the same key terms to identify eligible surveys with no available published reports. This approach was deemed the most effective method for creating the comprehensive overview presented in this review.

## Conclusion and recommendations for future NNSs

This review provides an overview of NNSs worldwide, which could inform policymakers about current practices of the use of national health surveys in other countries. This information can then be used as a baseline for establishing guidelines for future nutrition and health surveys. The finding of this review could potentially contribute to build upon existing NNSs to advance the understating of NNSs and lead to more informed decision-making that could improve the public health. Therefore, it would be helpful for future efforts to target the standardization of the methods to allow for more precise and informative data about the dietary intake. Here, we provide recommendations for potential future nutrition and health surveys based on an analysis of national surveys conducted in several countries:

1. The objectives of the survey need to be clearly defined. It is essential to decide whether the survey will focus on only nutrition status or a combination of health, nutrition, and environmental factors.
2. The geographic area and population groups that will be covered should be chosen with distinct reasoning. The survey needs to cover all administrative regions of the country, with a team assigned to each region for operation and management. The population groups are expected to have similar diets and mortality circumstances; surveys for adults, pregnant and lactating females, and children may need to be conducted separately [73]. In addition, streamlining age ranges using internationally recognized definitions such as those used by the World Health Organization can ensure clarity and comparability of survey results. By employing age categories such as 'child,' 'adolescent,' 'adult,' and 'elderly,' data collection and analysis can be enhanced across diverse studies and populations. It allows for the pooling of data for meta-analyses and the development of evidence-based guidelines and policies applicable to various populations and settings. Moreover, it is important to distinguish between sex and gender in data collection for nutrition research and the potential implications of conflating these terms [74].
3. A specific time frame must be decided with the help of community leaders to avoid limitations that may be placed upon the population, such as during holidays when people are away from their homes or have certain dietary restrictions. Seasonal weather should also be considered to avoid difficult travel.
4. Population and other data must be gathered. Before beginning the survey, it is essential to learn as much about the figures of population as possible, including the population size, characteristics (such as age, gender, ethnicity), socioeconomic data (such as education and income levels). Data can be retrieved from previous surveys, statistics, maps, and other anthropological information.
5. The sampling method and population size must be determined. The most practical design would be a cross-sectional design using sampling methodology appropriate for obtaining a representative sample of the population (a multistage stratified probability sample), potentially using data from the census bureau of the country for household selection and sample weighing.
6. What data to collect must be decided as determined by the objectives. Dietary and anthropometric data are the priority, but it is feasible to collect biochemical data from a subsample if sufficient resources are available. Samples collected need not necessarily be analysed for several biomarkers in the same time frame; analysis could be postponed until more resources are available. However, it is important to consider not only the cost of the analysis, but also the other expenses such as sample storage and management. An appropriate assessment of these factors alongside budgetary considerations confirms the overall feasibility of the survey.
7. Survey teams must be selected and trained. The teams must be carefully trained in data collection by the researchers prior to conducting the survey, but it is not necessary that they be health professionals. Teams should comprise people from the community who are fit and have a high level of education, as they will need to record the data accurately. Females are preferable for interviewing young children and other females [75]. The type of survey team chosen depends on the data to be collected. If children are surveyed, it is ideal to have at least two people: one to measure the child, and one to record the information. Additionally, an interviewer should be present. The number of teams required depends on the number of people to be visited within the time given to complete the survey and the size of the area covered.
8. There is no one-approach-fits-all when it comes to dietary assessment tools and national nutrition surveys. It is important to consider the survey objectives, feasibility of type of dietary tools (24 h recall, diaries, FFQ), validation of the chosen tool for the specific population, duration, portion size estimation, food composition databases, and available resources. In addition, it is recommended to take into consideration the advantages and weaknesses when employing 24-h dietary recalls, FFQ, or both. Combining 24 h recalls with FFQ will compensate for their limitations by providing a comprehensive and accurate assessment of dietary intake. However, strategic and innovative approaches are required to reduce the burden on participants and survey staff, such as using web-based systems, automated nutrient calculation, and digital technologies (mobile apps). Younger generations, particularly those born after 1981, are heavily involved in technology, so integrating digital tools like mobile apps and online interviews can enhance the accuracy and reliability of dietary assessments, providing a more tailored and engaging approach to data collection [76].
9. When designing or revising future NNS, it is important to consider the available budgets (government/ sponsors) and human resources. By taking into account the available budget, the feasibility, efficiency, and quality of the NNS can be confirmed, which will help in prioritization of key survey’s objectives within the allocated resources.

### Supplementary Information

**Supplementary Material 1.**

## Data Availability

The datasets used and/or analysed during the current study available from the corresponding author on reasonable request.
