# Supplementary material for: Cross-continental national nutrition surveys: a narrative review
Source: BMC Nutr. 2024 Apr 22;10:63. doi: 10.1186/s40795-024-00868-4 (PMC11034115; doi:10.1186/s40795-024-00868-4)
Supplement: Supplementary file 1 — Supplementary Material 1. [file 40795_2024_868_MOESM1_ESM.docx]

**Supplementary Table**

**Supplementary table:** Main objective of the national nutrition surveys according to country (n = 41)

| **Country** | **Full survey name** | **Survey’s objective** |
| --- | --- | --- |
| Canada | Canadian Health Measures Survey (CHMS)  Canadian Community Health Survey – Nutrition (CCHS) | To gather information that will help improve the prevention, diagnosis and treatment of illnesses and to promote the health and wellness of Canadians.  To gather information at the provincial level on the overall nutritional status of the Canadian population. |
| US | The National Health and Nutrition Examination Survey (NHANES) | To assess the health and nutritional status of adults and children in the United States. |
| UK | The National Diet and Nutrition Survey (NDNS) | To provide annual data about the nation’s dietary intake and nutritional status. |
| Greece | Hellenic National Nutrition and Health (HNNHS)  Greek National Diet and Health Survey (HYDRIA) | To assess nutritional intake, health status and various behaviors in the Greek population.  To assess the diet and health status of the adult population in Greece. |
| Poland | Multi-Centre National Population Health Examination Survey (WOBASZ) | To evaluate prevalence, control, treatment, and morbidity |
| Portugal | National Food and Physical Survey (IAN-AF) | To collect national information on food consumption and on physical activity and its relationship with health determinants, including socioeconomic determinants. |
| Spain | National Food Survey on Adults, Elderly People and Pregnant Women (ENALIA2) | To collect food consumption data and other information about eating habits and physical activity on adults, elderly and pregnant women. |
| Switzerland | The National Nutrition Survey (menuCH) | To determine what and how much people living in Switzerland eat and drink, when and where. |
| Turkey | Turkey Nutrition and Health Survey (TNHS) | To gather data on the nutritional status, physical activity level, and socioeconomic situations within the population of Turkey in order to plan programs and develop indicators. |
| Russia | Russian Longitudinal Monitoring Survey (RLMS) | To monitor the effects of Russian reforms on the health and economic welfare of households and individuals in the Russian Federation. |
| Czech Republic | Czech National Food Consumption Survey (SISP) | To deeply assess the population's dietary exposure |
| Finland | The National FINDIET Survey | To monitor dietary habits and nutrient intake of the adult Finnish population. |
| Hungary | Hungarian National Food Consumption Survey among Adults | To collect data on food consumption for assuring food safety. |
| Iceland | Icelandic National dietary Survey | To monitor diet development, nutrient intake, additives, and contaminants among citizens |
| Austria | Austrian Nutrition Survey | To evaluate of the nutrient intake of the Austrian Adults |
| Belgium | Belgium National Food Consumption Survey (BNFCS) | To evaluate the habitual food, energy and nutrient intake in the Belgian population and to compare these with recommendations from the national dietary guidelines. |
| Denmark | Danish National Survey of Diet and Physical Activity (DANSDA) | To monitor dietary intake and physical activity in a representative sample of the Danish population aged 4-80 years and provide data for surveillance of the food and nutrient intake and physical activity of the general population. |
| France | French Nutrition and Health Survey (ESTEBAN) | To describe food consumption, physical activity, sedentariness, and nutritional status and to estimate the prevalence of certain chronic diseases and vascular risk factors. |
| Germany | German National Nutrition Survey (NVS) | To evaluate food consumption and other aspects of nutritional behaviour of a representative sample of the German population |
| Ireland | National Adult Nutrition Survey (NANS) | To provide up-to-date quantitative, habitual food consumption data separately for all eating occasions over each of four days at the level of the individual and is suitable for a wide range of applications related to food safety and nutrition. |
| Italy | The Third Italian National Food Consumption Survey (INRAN-SCAI) | To identify the main dietary sources of nutrients in the diet of the population in Italy |
| Netherlands | Dutch National Food Consumption Survey (DNFCS) | To monitor the food consumption and intake of energy and nutrients of the general Dutch population |
| Japan | National Health and Nutrition Survey (NHNS) | To collect basic data on the health, nutrition and lifestyles of adults and children for comprehensive promotion of the nation’s health. |
| Mongolia | Mongolia National Nutrition Survey (NNS) | To determine the current nutrition and health status of the population, identifies changes, and provides background information and evidences for future activities to improve nutrition and health of Mongolians. |
| South Korea | The Korea National Health and Nutrition Examination Survey (KNHANES) | To assess the health and health-related behaviors of Korean population |
| China | China Health and Nutrition Survey (CHNS) | To develop a multipurpose longitudinal survey that would allow the group to examine a series of economic, sociological, demographic and health questions of interest to the Chinese Academy of Preventive Medicine and these scholars |
| India | National Nutrition Monitoring Bureau (NNMB) Survey | To assess the food and nutrient intakes at household and individual level, and to carry out anthropometry and clinical examination of individual to assess their nutritional status, on a continuous basis, on representative segments of the rural/urban/tribal population in the States, by adopting standard procedures and techniques; to evaluate ongoing national nutrition programmes to identify their strengths and lacunae and to recommend appropriate corrective measures. |
| Philippines | National Nutrition Survey (NNS) | To determine and evaluate the food intakes, nutrition and health status of Filipinos, and provide official statistics on food, nutrition and health situations of the country. |
| Singapore | National Nutrition Survey (NNS)  National Population Health Survey (NPHS) | To inform how the population’s eating habits have evolved over time and highlights areas of public health concern which require action and drives the development of health promoting nutrition policies, strategies and programmes.  To collect pertinent insights on demographics and health-related factors such as chronic diseases, exercise, health screening, mental well-being, nutrition and smoking. |
| Taiwan | Nutrition and Health Survey in Taiwan (NAHSIT) | To establish a long-term, stable and regular monitoring system, which can monitor the national health and nutrition status |
| Thailand | The Thai Food Consumption Survey (TFCS) | To evaluate food and nutrient consumption patterns and health status in the Thai population as a whole and to evaluate regional differences |
| Saudi Arabia | Saudi Health Interview Survey (SHIS)  National Survey of Health, Diet, Physical Activity and Supplements among Adults  World Health Survey Saudi Arabia (KSAWHS) | To collect data on health and demographic characteristics in order to assess the prevalence of several chronic conditions and identify their risk factors  To determine overall health status, nutritional perceptions, knowledge, behaviors, and shopping practice among the Saudi population  To provide up-to-date, timely and relevant information on Sustainable Development Goals health-related indicators and WHO indicators, framework programmatic indicators, and socio-demographic stratifiers. |
| Kuwait | Kuwait National Nutrition Survey (KNNS) | To provide regular and updated information on the nutritional status of the Kuwaiti population (children and adults) and the influencing factors. |
| United Arab Emirates | UAE World Health Survey (UAEWHS) | To achieve a world-class healthcare system by offering exceptional services delivered in a sustainable surrounding |
| Australia | National Health Survey (NHS)/National Nutrition and Physical Activity Survey (NNPAS) | To help address key health information gaps in nutrition and physical activity |
| New Zealand | New Zealand Adult Nutrition Survey (NZANS) | To evaluate the risk to New Zealanders from exposure to chemicals in foods. |
